# Proton pump inhibitor use and pancreatic risk: evidence from the UK biobank participants and animal experiments

**DOI:** 10.3389/fphar.2025.1673200

**Published:** 2025-10-08

**Authors:** Xin Gao, Zouhua Xu, Qingxie Liu, Chenchen Yuan, Xiaowu Dong, Xiaolei Shi, Qingtian Zhu, Keyan Wu, Hongwei Xu, Jiajia Pan, Guotao Lu, Weiming Xiao, Shengfeng Wang, Yaodong Wang

**Affiliations:** ^1^ Pancreatic Center, Department of Gastroenterology, Affiliated Hospital of Yangzhou University, Yangzhou University, Yangzhou, Jiangsu, China; ^2^ Yangzhou Key Laboratory of Pancreatic Disease, Institute of Digestive Diseases, The Affiliated Hospital of Yangzhou University, Yangzhou University, Yangzhou, Jiangsu, China; ^3^ Department of Gastroenterology, Kunshan Hospital of Traditional Chinese Medicine, Kunshan Affiliated Hospital of Yangzhou University, Kunshan, Jiangsu, China; ^4^ Suzhou Key Laboratory of Integrated Traditional Chinese and Western Medicine of Digestive Diseases, Kunshan Hospital of Traditional Chinese Medicine, Kunshan, Jiangsu, China; ^5^ Department of Epidemiology and Biostatistics, School of Public Health, Peking University Health Science Center, Beijing, China; ^6^ Key Laboratory of Epidemiology of Major Diseases (Peking University), Ministry of Education, Beijing, China

**Keywords:** proton pump inhibitors, pancreatic disease incidence, drug safety, prospective cohort, health sciences

## Abstract

**Introduction:**

Proton pump inhibitors (PPIs) are widely prescribed for gastrointestinal disorders and are often used empirically in patients with pancreatic disease, yet their long-term impact on pancreatic health remains unclear. We evaluated whether regular PPI use is associated with risks of acute pancreatitis (AP), chronic pancreatitis (CP), and pancreatic cancer (PC).

**Methods:**

We analyzed 489,394 UK Biobank participants aged 38–73 years, comparing regular PPI users with non-users and with histamine-2 receptor antagonist (H_2_RA) users as an active comparator. Associations with incident pancreatic outcomes were estimated using Cox regression models, landmark analysis, and propensity score matching, supplemented by multiple sensitivity analyses, including stratified/interaction analyses, E-values, time-varying exposure models with immortal-time correction, dfbeta residuals correction, stricter follow-up with Firth penalization, full-cohort multivariable modeling, and alternative matching (disease risk score 1:1, entropy balancing). Complementary *in vivo* experiments used a cerulein-induced acute pancreatitis mouse model to examine the effects of short- and long-term PPI administration on pancreatic inflammation and histopathology.

**Results:**

In primary analyses, regular PPI use showed a time-dependent association with acute pancreatitis. However, this association was not robust: multiple sensitivity analyses indicated instability of the finding. Experimental validation in mice demonstrated that neither short-term nor long-term PPI administration altered pancreatic inflammation or histopathological damage in the cerulein-induced model.

**Discussion:**

Integrating large-scale cohort data with experimental evidence, our findings suggest that regular PPI use does not meaningfully influence the risk of acute pancreatitis, chronic pancreatitis, or pancreatic cancer.

## 1 Introduction

Proton pump inhibitors (PPIs), since their introduction in the 1980s, have rapidly replaced histamine type 2 receptor antagonists (H_2_RAs) (Savarino et al., 2017) and have become one of the most commonly used pharmaceuticals worldwide for the treatment of clinical acid-related disorders (Gracie and Ford, 2016). Commonly used PPIs include omeprazole, lansoprazole, pantoprazole, rabeprazole, and esomeprazole. They suppress gastric acid secretion by inhibiting the enzyme hydrogen-potassium adenosine triphosphatase (H^+^-K^+^-ATPase) in gastric parietal cells. Over time, their indications have expanded beyond the initial treatment of peptic ulcers and gastroesophageal reflux disease to other gastric acid-related diseases (Malfertheiner et al., 2017).

The mean rate of overprescription for antisecretory drugs, mainly PPIs, among ambulatory and hospitalized patients is estimated to be as high as 50%–60% (Savarino et al., 2017), inevitably bringing unpredictable side effects of gastrointestinal and non-gastrointestinal conditions, such as pancreatitis (Malfertheiner et al., 2017; Nardino et al., 2000; Reid et al., 2012; Savarino et al., 2016). Given these concerns, particular attention has been drawn to their role in pancreatic disorders, where clinical practice and guideline recommendations diverge. Despite their frequent empirical use in the management of pancreatitis (Murata et al., 2015; Ma et al., 2020; Zhang et al., 2021) in practical clinical applications, PPIs are not endorsed by current guidelines (Gardner et al., 2020; Working Group IAP/APA Acute Pancreatitis Guidelines, 2013; Yo et al., 2015), largely owing to the absence of supporting evidence from large population-based trials. Notably, omeprazole was reported to have the highest level of evidence for its association with acute pancreatitis (AP) in the systematic review of case reports of drug-induced pancreatitis (Zhang et al., 2021; Gardner et al., 2020). However, both the definitive diagnosis and pathogenic mechanism of drug-induced pancreatitis remain poorly defined (Jones et al., 2015).

Evidence from previous studies on the relationship between PPI use and AP is inconsistent. Youssef et al. (2005) and Kathi et al. (2020) reported a single case of omeprazole-induced pancreatitis. A case-control network study on drug-induced AP morbidity in Sweden found that PPI use was significantly associated with AP (Blomgren et al., 2002). Other cohort studies reported no significant increase in the risk of AP in PPI users (Eland et al., 2000; Douros et al., 2013). Previous *in vivo* (Burdan et al., 2000) and *in vitro* cellular experiments (Cai et al., 2007) have concluded that omeprazole has no clear potential to increase the pathogenicity of AP.

Overall, these studies remain inconclusive in defining the association between PPI use and the risk of subsequent pancreatic disease. Previous studies were heterogeneous due to factors such as insufficient sample size (fewer than 500 research subjects or cases (Blomgren et al., 2002; Eland et al., 2000; Douros et al., 2013)), geographical limitations of the research, variations in research designs, and lack of consideration of PPI use duration Using data from the UK Biobank, we conducted large-scale population-based analyses to address the primary relationship between PPI administration and AP. To minimize residual confounding in large-scale epidemiological analyses and provide more robust evidence for the observed associations, we conducted a series of sensitivity analyses. In parallel, we conducted animal experiments to provide additional validation and to gain preliminary insights into the potential association between PPI use and pancreatic injury.

## 2 Methods

### 2.1 Study population

This study utilized data from the UK Biobank, a large-scale biomedical database and research resource containing health data of more than 500,000 participants. Individuals were recruited between 2006 and 2010. Participants underwent a repeat baseline assessment visit, which included obtaining information on their health and lifestyle, collected through an electronic questionnaire and a brief verbal interview. Repeat assessments were scheduled every 2–3 years during follow-up (Collins, 2012). The UK Biobank study received ethical approval from the National Information Governance Board for Health and Social Care in England and Wales, the Community Health Index Advisory Group in Scotland, and the North West Multi-Centre Research Ethics Committee. All participants provided written informed consent. Data access for this study was granted through the UK Biobank Access Management System (Application ID 69476).

For the exposed population, the date of the first recorded regular PPI or H_2_RA use was defined as the inclusion time. For non-users, the inclusion time was defined as the date of their first health assessment visit. Exclusion criteria included: (1) missing information in any covariate; (2) uncertain answers (e.g., “do not know, do not want to answer”) to key questions; (3) a history of pancreatic disorders and open pancreatic surgery prior to enrollment; (4) simultaneous use of both PPI and H_2_RA.

### 2.2 Assessment of medication use

Participants were classified into three groups according to medication use: regular PPI users, regular H_2_RA users, and control participants with no history of PPI or H_2_RA (see Supplementary Table 1 for detailed codes). Participants were classified as regular PPI or H_2_RA users if they met the following conditions: (1) they reported taking any commonly prescribed or over-the-counter medications in the touchscreen questionnaire; and (2) when asked by the trained interviewer, “In the touchscreen questionnaire, you said you are taking regular prescription medications. Can you tell me what these are?” If no data was retrieved, the interviewer confirmed with the participants to determine if this was the case. The interviewer used the “search facility” or “free-text” to enter a medication. Drug information on prescription medications taken by participants weekly, monthly, or for 3 months would be recorded, whereas short-term medications, such as a 1-week course of antibiotics for an infection or analgesics taken 2 days ago for a migraine, would not be included. The PPIs recorded included omeprazole, lansoprazole, pantoprazole, rabeprazole, and esomeprazole, while the H_2_RAs recorded were ranitidine, cimetidine, famotidine, and nizatidine.

### 2.3 Outcome and covariates

In this prospective study, the incidence of pancreatic disease—including acute pancreatitis (AP), chronic pancreatitis (CP), and pancreatic cancer (PC)—was defined as the study endpoint (see Supplementary Table 2 for detailed codes). In the cohort analysis of patients with AP, covariates included socio-demographic factors (sex, age, ethnicity, Townsend deprivation index), lifestyle factors (body mass index (BMI), smoking status, alcohol intake frequency), and relevant clinical history. The latter comprised diagnosis of diabetes, disorders of lipoprotein metabolism and other lipidemias, cholelithiasis, significant surgical history (therapeutic endoscopic retrograde operations within 1 month before diagnosis), and medication history (drug types that were reported to possibly cause pancreatitis). In particular, drugs classified as Class Ia—defined according to Wolfe et al.‘s research (Wolfe et al., 2020) as agents with at least five human case reports and a positive rechallenge indicating possible drug-induced pancreatitis—were included as potential confounders. Other medications considered as covariates are listed in Supplementary Table 1. For individuals who had been exposed to suspected pathogenic drugs during the same period, this covariate was recorded as the total number of medication types. The covariates involved in the analysis for patients with CP and PC are detailed in Supplementary Material (Supplementary Text Material 1).

### 2.4 Animals and ethical approval

All animal procedures were approved by the Experimental Animal Ethics Committee of Yangzhou University, and animals were cared for according to the principles of laboratory animal care (NIH publication number 85Y23, revised 1996). Male ICR mice (four to six weeks, 20–22 g) were purchased from the Yangzhou University Model Animal Center (Yangzhou, China). Animals were maintained under specific pathogen-free (SPF) conditions with a 12-h light/dark cycle, at 23 °C ± 2 °C and 45% relative humidity, with ad libitum access to standard chow and water. All animal experiments complied with the principles of replacement, refinement, and reduction (The 3Rs).

### 2.5 Reagents and dosing regimens

Omeprazole and lansoprazole were purchased from Shanghai Aladdin Biochemical Technology Co., Ltd. (Shanghai, China) and dissolved in dimethylsulphoxide (DMSO) at 10 mg/mL, then diluted to a final gavage volume of 200 μL with 0.5% sodium carboxymethyl cellulose (CMC-Na). To model both chronic clinical exposure and acute high-level drug administration, two dosing regimens were calculated using body-surface–area (BSA) allometric scaling (Nair and Jacob, 2016). Long-term low-dose treatment was used to approximate the human-equivalent therapeutic range after adjusting for interspecies metabolic differences, thereby reflecting chronic exposure conditions. In contrast, short-term high-dose treatment was applied to mimic acute supratherapeutic exposure, a strategy commonly adopted in pharmacological and toxicological studies to induce measurable biological responses within a limited timeframe (Burdan et al., 2000; Buckley and Dorato, 2009).

Accordingly, male ICR mice were randomly assigned to four groups and treated by oral gavage: (1) normal group, receiving 200 μL 0.5% CMC-Na (long-term: 35 days; short-term: 3 days); (2) AP modeling control group, receiving the same CMC-Na regimen; (3) omeprazole group, receiving 3 mg/kg/day for 35 days (long-term) or 30 mg/kg/day for 3 days (short-term); and (4) lansoprazole group receiving 5 mg/kg/day for 35 days (long-term) or 50 mg/kg/day for 3 days (short-term). Body weight was measured prior to each gavage to ensure accurate dosing. Cerulein (Cat. Pep04059, Cae) was obtained from Nanjing Peptide (Nanjing, China). All groups, except the normal control group, were induced in the mild AP model by intraperitoneal injection of Cae (10 μg/kg, interval of 1 h, seven times), while equivalent volumes of PBS was used in the normal group (specific groupings and experimental flow are shown in Supplementary Figure 5A). All mice were euthanized 12 h after the first injection of Cae under deep anesthesia with intraperitoneal pentobarbital. Blood samples were stored at room temperature for 30 min and centrifuged at 4,000 *g* at 4 °C for 15 min to isolate serum, which was analyzed for amylase and lipase according to kit instructions. Pancreatic tissues were harvested immediately and fixed for histological analysis. Paraffin sections of the pancreas were stained with hematoxylin and eosin. Three pathologists who were blinded to the experimental treatment scored the degree of pancreatic injury by light microscopy and evaluated the severity of edema, inflammation, and necrosis, as described previously, with detailed scoring criteria available in reference (Pan et al., 2017).

### 2.6 Statistical analysis

A total of 489,394 participants were left as the primary population after exclusion, including 54,453 regular PPI users, 8,293 regular H_2_RA users, and 426,648 users who took neither PPI nor H_2_RA (Supplementary Figure 1; Supplementary Table 3). For descriptive statistics, categorical variables are described as percentages and compared using the chi-square test or Fisher’s exact test. Continuous variables are expressed as medians with interquartile ranges (IQRs) and compared using the Wilcoxon rank-sum test (Mahmud et al., 2022).

**FIGURE 1:**
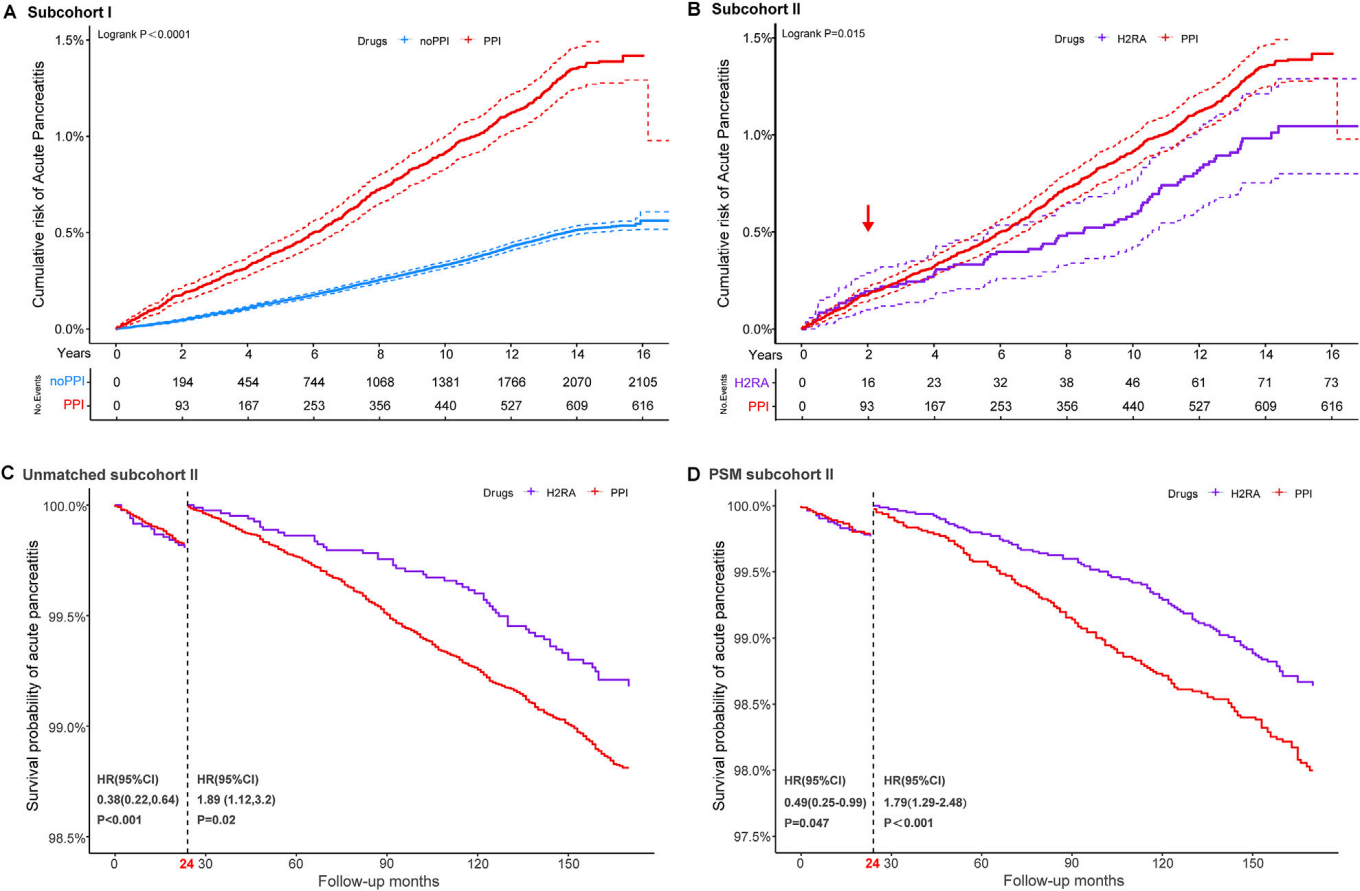
Cumulative probability and landmark analysis of AP. Subcohort I: regular PPI users versus non-PPI users; subcohort II: regular PPI users versus regular H_2_RA users. Kaplan–Meier curves illustrate the cumulative incidence of AP in regular proton pump inhibitor (PPI) users, regular histamine-2 receptor antagonists (H_2_RAs) users, and those without regular medications **(A,B)**. Landmark analyses of AP events occurring within and beyond 2 years of follow-up in the unmatched **(C)** and propensity score-matched subcohorts **(D)**.

We plotted crude cumulative incidence curves for pancreatic outcomes across medication groups and visually assessed proportional hazards assumption (Figure 1; Supplement Figure 3). Given that PPIs and H_2_RAs are prescribed for similar indications, H_2_RAs were chosen as an active comparator to reduce potential confounding by indication (Abrahami et al., 2022).

Cox regression models were used to estimate crude and adjusted hazard ratios (HRs) with 95% confidence intervals (CIs) for each endpoint. In the primary analysis, univariate Cox regressions were performed on relevant variables to evaluate whether medication use was an important pathogenic factor in the two subcohorts, with covariates including age, sex, race, index of multiple deprivations, BMI, alcohol intake frequency, smoking status, hyperlipidemia, cholelithiasis, recent therapeutic endoscopic retrograde operations, and types of relative drugs. To better control for confounding factors, propensity scores were estimated using logistic regression, representing the predicted probability of receiving different treatment conditional on the covariates listed above. Balance between treated and control groups was assessed using standardized mean differences (SMD). Following propensity score matching, comparative analyses were conducted to evaluate differences in pancreatic disease incidence between the exposure cohorts. Considering that the cumulative survival curves of AP in the PPI versus H_2_RA subcohorts crossed over (Figure 1B), we conducted a landmark analysis at 24 months after registration to evaluate the association between PPI use and AP in patients who remained uncensored beyond this threshold (Maeng et al., 2014).

Sensitivity analyses were conducted as follows: a) Stratified and interaction analysis: In addition to stratified analyses of individual PPIs (omeprazole, rabeprazole, esomeprazole, lansoprazole, and pantoprazole), further stratification was performed by key demographic and clinical variables, including sex, age, races, BMI, the index of multiple deprivation and other drug types. Interaction analyses were subsequently conducted to examine whether the associations between drug exposure and AP risk were modified by these variables. b) E-value analyses: E-values were calculated as E = HR + sqrt [HR × (HR−1)] describe the extent to which unmeasured confounders need to correlate with the exposure and the outcome on the HR scale. E-values were calculated using 1/HR rather than HR (VanderWeele and Ding, 2017) for factors negatively associated with disease outcomes. c) Time-varying exposure Cox model: We applied time-varying Cox proportional hazards models to account for changes in drug use over follow-up. This approach allowed participants to transition between exposure states, yielding risk estimates based on contemporary exposure. To improve resolution, models with more than two follow-up time splits were also fitted (Andersen and Gill, 1982). d) Correcting immortal time bias: The inclusion stage of participants’ exposure to propensity score matching (PSM) was restricted to ensure that exposure classification corresponded with the time of cohort entry (Yu and Suissa, 2023). e) dfbeta Residuals: We identified influential observations using dfbeta residuals and repeated the Cox regression after excluding influential cases to assess robustness of the estimates. f) Multivariate Cox regression in the full cohort: A direct multivariable Cox regression analysis was conducted in the entire population to assess the AP endpoint. g) Limiting the number of follow-up visits step-by-step: Robustness was further evaluated by restricting the analysis to participants with ≥2, ≥3, and ≥4 follow-up visits. As stricter follow-up requirements reduced sample size and events, Firth’s penalized Cox regression was applied to mitigate sparse data bias and stabilize estimates (Heinze and Schemper, 2002; Zegard et al., 2021). Strata with fewer than 10 observed events were excluded to avoid unreliable estimates. h) Alternative matching methods: We further applied disease risk score (DRS) 1:1 matching and entropy balancing matching (EBM) as alternative approaches to test robustness against matching method choice.

For the analysis of animal experimental data, differences between two groups were assessed using unpaired Student’s t-test, with p < 0.05 considered statistically significant. Comparisons among multiple groups were conducted using one-way analysis of variance (ANOVA) followed by Tukey’s post hoc test. Histopathological scores were analyzed using a nonparametric test.

All statistical analyses and graphical presentations were performed using SAS (version 9.4), R (version 4.1.2), GraphPad Prism (version 8.0.2), and Origin (version 9.8).

## 3 Results

### 3.1 Baseline characteristics and cumulative incidence curves

As shown in Table 1 and Supplementary Figure 1, the PSM-adjusted cohorts comprised 52,324 regular PPI users and 52,324 non-PPI users in subcohort I (regular PPI versus non-PPI users), and 8,293 regular PPI users and 8,293 regular H_2_RA users in subcohort II (regular PPI versus regular H_2_RA users).

**TABLE 1:** Baseline characteristics and standardized mean differences (SMD) before and after propensity score matching (PSM) in different cohorts in the UK Biobank.

Variables	Subcohort I: PPI vs*.* non-PPI cohort						Subcohort II: PPI vs*.* H_2_RA cohort					
	Unmatched cohort			PSM cohort			Unmatched cohort			PSM cohort		
	PPI	neither	SMD (%)	PPI	neither	SMD (%)	PPI	H_2_RA	SMD (%)	PPI	H_2_RA	SMD (%)
	(N = 54,453)	(N = 426,648)		(N = 52,324)	(N = 52,324)		(N = 54,453)	(N = 8,293)		(N = 8,293)	(N = 8,293)	
Age (median (IQR), years)	62.0 (56.0,67.0)	58.0 (50.0,63.0)	55.8	62.0 (56.0,67.0)	63.0 (57.0,66.0)	1.1	62.0 (56.0,67.0)	60.0 (52.0,65.0)	32.9	60.0 (52.0,65.0)	60.0 (52.0,65.0)	0.3
Sex = male (%)	24,681 (45.3)	194,198 (45.5)	0.4	24,677 (45.3)	24,801 (45.6)	0.5	24,681 (45.3)	3,749 (45.2)	0.2	3,749 (45.2)	3,704 (44.7)	1.1
Race = white (%)	51,935 (95.4)	402,463 (94.3)	4.7	51,929 (95.4)	51,915 (95.3)	0.1	51,935 (95.4)	7,857 (94.7)	2.9	7,857 (94.7)	7,850 (94.7)	0.4
Index of multiple deprivation (median (IQR))	−1.94 (−3.55,1.02)	−2.19 (−3.67,0.42)	9.9	−1.94 (−3.55,1.02)	−1.93 (−3.50,0.90)	0.3	−1.94 (−3.55,1.02)	−1.76 (−3.43,1.32)	6.3	−1.72 (−3.44,1.35)	−1.76 (−3.43,1.32)	0.2
BMI (median (IQR), kg/m^2^)	28.3 (25.6,31.7)	26.5 (24.0,29.6)	37.7	28.3 (25.6,31.7)	28.1 (25.2,31.8)	0.2	28.3 (25.6,31.7)	28.0 (25.3,31.4)	5.8	28.1 (25.3,31.6)	28.0 (25.3,31.4)	2.1
Alcohol intake frequency (%)		19.7			0.6			8.4			1.6	
never	6,342 (11.6)	31,721 (7.4)		6,340 (11.6)	6,241 (11.5)		6,342 (11.6)	858 (10.3)		858 (10.3)	890 (10.7)	
≤3 times a month	14,512 (26.7)	94,576 (22.2)		14,510 (26.6)	14,563 (26.7)		14,512 (26.7)	2043 (24.6)		2043 (24.6)	2070 (25.0)	
1–4 times a week	23,893 (43.9)	212,319 (49.8)		23,892 (43.9)	23,925 (43.9)		23,893 (43.9)	3,690 (44.5)		3,690 (44.5)	3,643 (43.9)	
Daily or almost daily	9,706 (17.8)	88,032 (20.6)		9,705 (17.8)	9,718 (17.8)		9,706 (17.8)	1702 (20.5)		1702 (20.5)	1,690 (20.4)	
Smoking status (%)			17.8			0.5			12.7			2
Never	26,134 (48.0)	238,675 (55.9)		26,134 (48.0)	26,105 (47.9)		26,134 (48.0)	3,798 (45.8)		3,798 (45.8)	3,834 (46.2)	
Previous	22,925 (42.1)	143,456 (33.6)		22,920 (42.1)	22,868 (42.0)		22,925 (42.1)	3,331 (40.2)		3,331 (40.2)	3,256 (39.3)	
Current	5,394 (9.9)	44,517 (10.4)		5,393 (9.9)	5,474 (10.1)		5,394 (9.9)	1,164 (14.0)		1,164 (14.0)	1,203 (14.5)	
Diabetes (%)	5,478 (10.1)	19,449 (4.6)	21.3	5,475 (10.1)	5,327 (9.8)	0.9	5,478 (10.1)	672 (8.1)	6.8	672 (8.1)	701 (8.5)	1.3
Hyperlipidemia (%)	10,108 (18.6)	49,928 (11.7)	19.2	3,113 (5.7)	3,106 (5.7)	0.1	10,108 (18.6)	1,211 (14.6)	10.7	1,211 (14.6)	1,212 (14.6)	<0.1
Cholelithiasis (%)	3,113 (5.7)	12,700 (3.0)	13.5	10,105 (18.6)	10,227 (18.8)	0.6	3,113 (5.7)	445 (5.4)	1.5	445 (5.4)	466 (5.6)	1.1
Therapeutic endoscopic retrograde operations (%)	439 (0.8)	1,578 (0.4)	5.7	439 (0.8)	403 (0.7)	0.8	439 (0.8)	66 (0.8)	0.1	66 (0.8)	62 (0.7)	0.6
Types of relative drugs (%)			43.2			2.5			5.4			<0.1
0	27,831 (51.1)	305,454 (71.6)		27,831 (51.1)	28,001 (51.4)		27,831 (51.1)	4,371 (52.7)		4,371 (52.7)	4,373 (52.7)	
1–3 types	26,486 (48.6)	121,091 (28.4)		26,486 (48.6)	26,373 (48.4)		26,486 (48.6)	3,916 (47.2)		3,916 (47.2)	3,914 (47.2)	
≥4 types	136 (0.2)	103 (0.0)		130 (0.2)	73 (0.1)		136 (0.2)	6 (0.1)		6 (0.1)	6 (0.1)	

SMD: standardized mean differences.

Table 1 lists baseline characteristics of participants before and after PSM. Prior to matching, regular PPI users tended to be older, had higher BMI, and exhibited a higher prevalence of diabetes and hyperlipidemia compared with controls. After PSM, covariates were well balanced across groups (Table 1; Supplementary Figure 2) with the maximum standardized difference reduced from 55.8% to 2.1%.

Figure 1 and Supplementary Figure 3 show the cumulative incidence curves for AP, CP and PC in the different cohorts using a log-rank test. In subgroup I, the incidence of pancreatic events was consistently higher among PPI users than non-users throughout the 16-year follow-up, with statistically significant differences observed for AP, CP, and PC. In subcohort II, the log-rank test suggested that the relationship between regular PPI use and the incidence of AP showed significant temporal heterogeneity compared to H_2_RA users, but a crossover of the two curves at 24 months could not be neglected.

### 3.2 Main data analysis shows that regular use of PPIs influences AP susceptibility

**FIGURE 2:**
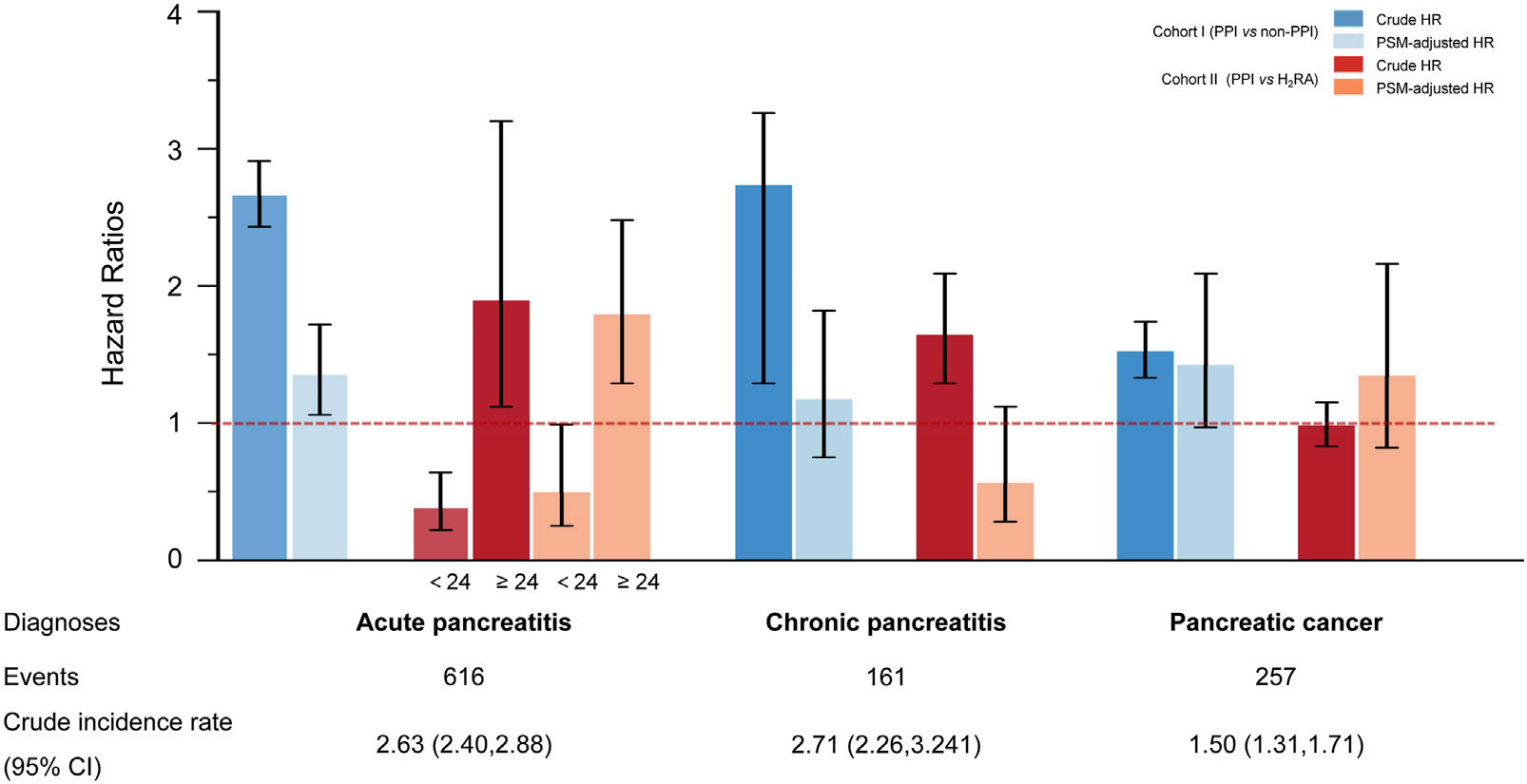
Risk of pancreatic disorders with the regular use of proton pump inhibitors in different cohorts Pancreatic disorders include acute and chronic pancreatitis and pancreatic cancer. The crude incidence rate is reported per 1,000 person-years. In subcohort I (regular PPI users versus non-PPI users), data are presented as crude hazard ratios (crude HRs) obtained from univariate Cox regressions and propensity score matching-adjusted hazard ratios (PSM-adjusted HR). Error bars indicate 95% confidence intervals (CIs). In subcohort II (regular PPI users versus regular H_2_RA users), the risk of acute pancreatitis (AP) was analyzed in two strata according to whether medication duration was less than or greater than 24 months.

Figure 2 and Supplementary Figure 4 illustrate the association between regular PPI use and pancreatic outcomes across cohorts.

In subcohort I, regular PPI use was consistently associated with an elevated risk of AP compared with non-regular PPI use, regardless of which model was applied. Compared to the result of crude univariate Cox regression (crude HR: 2.66, 95% CI: 2.43–2.91, p < 0.001), the association was attenuated (HR: 1.95, 95% CI: 1.71–2.22, p < 0.001) but remained significant after multivariable adjustment.

In subcohort II, smoking status, diabetes, gallstone disease, short-term endoscopic retrograde operation history, and concomitant medication remained independent risk factors for pancreatic endpoint events, even when compared with the use of H_2_RAs (Supplementary Table 4). Considering the significant crossover of the cumulative incidence curves at 24 months (Figure 1B), a landmark analysis was conducted. As a result, participants taking long-term PPI medication achieved a protective effect against AP within the first 2 years of follow-up compared to those taking H_2_RAs. However, once the follow-up period exceeded 2 years, an increased risk of AP was observed in participants who regularly received PPIs compared with H_2_RA users. These findings persisted after 1:1 PSM.

Across all models and cohorts, no significant association was observed between regular PPI use and CP or PC (Figure 2; Supplementary Figure 4).

### 3.3 Instability of associations in multiple sensitivity analyses

Several sensitivity analyses were performed to verify the stability of the main results (Tables 2, 3; Supplementary Tables 5–10). The outcome remained stable only for omeprazole after stratifying the specific drugs (Table 2; Supplementary Table 5a-5b), with the association between other drugs and the risk of AP no longer significant. Subgroup analyses (Table 3; Supplementary Table 5c) showed that effect directions were broadly consistent but significance became unstable, and most interaction terms were not significant after PSM. Notably, a significant effect modification by age was observed in subcohort II after PSM, with the highest risk detected among older participants with PPI use exceeding 2 years. Approximately 50% of E-value confidence intervals (CIs) overlapped with 1 (Supplementary Table 6) (VanderWeele and Ding, 2017). Time-varying Cox models (Table 2) yielded results consistent in direction with the main analysis but lost statistical significance, and further increasing follow-up splits produced unstable estimates with wide confidence intervals. The results were generally consistent with the main analysis after correction for immortal time bias (Supplementary Table 7) and expansion to the original study population (distribution shown in Table 2; Supplementary Tables 3, 8). However, when analyses were restricted by the number of follow-up visits, the association between regular PPI use and AP onset became markedly unstable (Table 2). Table 2 presents the newly defined populations with different follow-up visit requirements, showing considerable variation in statistical significance. Given the pronounced fluctuation in HRs, we applied Firth’s penalized partial likelihood correction to Cox regression models where convergence was achieved, to improve model stability (Supplementary Table 9). Using alternative matching strategies (DRS 1:1 and EBM, Supplementary Table 10), overall results remained consistent with the main analysis, though short-term effects lost significance under DRS but remained significant under EBM, while long-term risks were confirmed by both methods. Across all cohorts and analytical approaches, the associations between PPI use and AP risk remained inconsistent and insufficiently robust, underscoring substantial uncertainty both for overall exposure and for specific PPI subtypes.

**TABLE 2:** Sensitivity analysis results.

Methods of analysis and medication	Events	Person-years	Crude incidence rate	Subcohort I: PPI vs*.* non-PPI cohort		Subcohort II: PPI vs*.* H_2_RA cohort		
			(95% CI)	Crude HR (95% CI)	PSM-adjusted HR (95% CI)	Administration time	Crude HR (95% CI)	PSM-adjusted HR (95% CI)
1) Stratified analysis								
Omeprazole	399	90.45	2.36 (2.12, 2.63)	**2.39 (2.15, 2.65)**	**1.44 (1.27, 1.64)**	<2 years	0.72 (0.49, 1.05) ^△^	**0.43 (0.20, 0.92)**
						≥2 years	0.99 (0.84, 1.17) ^△^	**1.50 (1.10, 2.06)**
Rabeprazole	18	128.58	3.09 (1.83, 4.89)	**3.10 (1.95, 4.93)**	**1.81 (1.13, 2.88)**	<2 years	1.88 (0.26, 13.52) ^△^	—
						≥2 years	1.57 (0.97, 2.54) ^△^	2.41 (0.89, 6.51) ^△^
Esomeprazole	24	77.41	1.86 (1.19, 2.77)	**1.87 (1.25, 2.79)**	1.08 (0.72, 1.61) ^△^	<2 years	1.27 (0.47, 3.44) ^△^	—
						≥2 years	0.82 (0.53, 1.28) ^△^	1.39 (0.57, 3.38) ^△^
Lansoprazole	214	94.39	2.36 (2.05, 2.72)	**2.38 (2.07, 2.74)**	**1.40 (1.21, 1.63)**	<2 years	0.83 (0.55, 1.26) ^△^	1.11 (0.48, 2.56) ^△^
						≥2 years	1.07 (0.90, 1.27) ^△^	1.16 (0.78, 1.73) ^△^
Pantoprazole	18	142.21	3.42 (2.02, 5.40)	**3.43 (2.16, 5.46)**	**2.00 (1.25, 3.19)**	<2 years	2.15 (0.68, 6.77) ^△^	4.17 (0.57, 30.77) ^△^
						≥2 years	1.54 (0.92, 2.57) ^△^	2.01 (0.64, 6.3) ^△^
PPI								
**2) Time-varying exposure Cox model**				**2.65 (2.42, 2.9)**	**1.73 (1.52, 1.97)**	<2 years	**0.44 (0.24, 0.83)**	0.53 (0.21, 1.33)
						≥2 years	**1.45 (1.10, 1.92)**	**1.80 (1.29, 2.52)**
**Time-varying exposure Cox model with more than 2 follow-up time splits**				**3.70 (2.74, 4.99)**	**2.27 (1.49, 3.44)**	<2 years	0.27 (0.03, 2.96)	—
						≥2 years	4.36 (0.59, 32.04)	8.2 (0.87, 76.8)
**3) Correction for immortal time bias**				—	**1.76 (1.55, 2.00)**	<2 years	—	0.49 (0.24, 0.99)
						≥2 years	—	**1.79 (1.29, 2.48)**
**4) dfbeta residuals**				**2.57 (2.35, 2.82)**	**1.85 (1.63, 2.10)**	<2 years	0.56 (0.30, 1.04)	**1.62 (1.22, 2.16)**
						≥2 years	0.53 (0.26, 1.11)	**1.48 (1.13, 1.94)**

	No. of different populations							
	PPI	non-PPI	H_2_RA					
**5) Expansion to whole population**	54,453	426,648	8,293	**1.83 (1.67, 2.01)**	—	<2 years	**0.52 (0.30, 0.91)**	—
						≥2 years	**1.42 (1.07, 1.87)**	
**6) Follow-up times ≥2**	12,255	61,166	1,261	**3.72 (2.75,5.02)**	**2.26 (1.50, 3.41)**	<2 years	0.37 (0.05, 2.85)	—
						≥2 years	4.90 (0.68, 35.51)	7.48 (0.95, 59.22)
**Follow-up times ≥3**	2,551	10,966	222	**4.95 (2.37, 10.31)**	**8.74 (1.85, 41.35)**	<2 years	—	—
						≥2 years	—	**1.79 (1.58, 2.03)**
**Follow-up times ≥4**	123	796	0	18.48 (0.90, 380)	—		—	

Results in bold represent p < 0.05. Crude incidence rate per 1, 000 person-years. △: The upper or lower limits of the confidence interval for the E-value include 1. The following sensitivity analyses were conducted: stratified analysis with specific drug (1), E-value analyses (△), Time-varying exposure Cox model (2), correction for immortal time bias (3) and dfbeta residuals (4). Multivariate Cox regression analysis of the full population queue (5) gradually limited the number of follow-up visits (6).

**TABLE 3:** Stratified and Interaction Analyses of Key Variables in cohorts after PSM.

Variable	Subcohort I: PPI vs*.* non-PPI PSM cohort			Subcohort II: PPI vs*.* H_2_RA PSM cohort					
				Administration time <2 years			Administration time ≥2 years		
	HR (95% CI)	*P* Value	*P* For interaction	HR (95% CI)	*P* Value	*P* For interaction	HR (95% CI)	*P* Value	*P* For interaction
Sex			0.22			0.25			0.8
Female	1.62 (1.36, 1.94)	<0.001		0.36 (0.15, 0.87)	0.27		1.85 (1.17, 2.9)	0.008	
Male	1.9 (1.59, 2.28)	<0.001		0.93 (0.27, 0.27)	0.9		1.7 (1.06, 2.71)	0.03	
Age			0.34			0.48			0.03
≤50	2.43 (1.49, 3.96)	<0.001		0.47 (0.47, 5.2)	0.54		1.13 (0.55, 2.31)	0.75	
50–67	1.77 (1.51, 2.07)	<0.001		0.6 (0.26, 1.35)	0.22		1.65 (1.11, 2.46)	0.01	
>67	1.65 (1.3, 2.09)	<0.001		0.28 (0.05, 1.54)	0.14		5.32 (1.82, 15.56)	0.002	
Races			0.9			0.11			0.99
White	1.76 (1.54, 2)	<0.001		0.52 (0.25, 1.07)	0.08		1.72 (1.24, 2.39)	0.001	
Other	1.68 (0.93, 3.04)	0.09		—	—		—	—	
BMI			0.06			0.71			0.85
<24	2.7 (1.77, 4.13)	<0.001		0.23 (0.02, 2.22)	0.2		0.05 (0.84, 5.16)	0.11	
24–28	1.79 (1.38, 2.31)	<0.001		0.47 (0.13, 1.74)	0.25		1.93 (0.98, 0.98)	0.06	
≥28	1.59 (1.36, 1.85)	<0.001		1.74 (0.26, 1.63)	0.36		1.64 (1.09, 2.47)	0.02	
Index of multiple deprivation			0.74			0.73			0.9
Low	1.94 (1.45, 2.6)	<0.001		1.04 (0.18, 5.83)	0.97		1.9 (0.85, 4.26)	0.12	
Medium	1.72 (1.45, 2.04)	<0.001		0.41 (0.14, 1.23)	0.11		1.84 (1.2, 2.81)	0.005	
High	1.67 (1.31, 2.13)	<0.001		0.6 (0.2, 1.79)	0.36		1.56 (0.81, 2.98)	0.18	
Other drug type			0.96			0.7			0.86
0	1.77 (1.45, 2.15)	<0.001		0.64 (0.23, 1.82)	0.41		1.74 (1.07, 2.82)	0.03	
1–3 types	1.71 (1.45, 2.02)	<0.001		0.48 (0.18, 1.26)	0.14		1.77 (1.14, 2.75)	0.01	
≥4 types	2.12 (0.22, 20.36)	0.52		—	—		—	—	

Stratified analyses were conducted in the matched cohorts, with pre-matching results shown in Supplementary Table 5c.

### 3.4 PPI treatment did not influence AP susceptibility in mice

The variable results of the sensitivity analysis prompted us to conduct additional animal experiments. We administered low-dose Cae to induce mild AP in ICR mice, with PBS as control subsequent development, after a defined period of gavage treatment, as shown in the experimental flowchart (Supplementary Figure 5a). Compared with the normal group, the Cae-induced AP model group showed significantly elevated serum amylase and lipase activities (Supplementary Figures 5C,G), as well as pancreatic edema, inflammatory infiltration, and acinar cell necrosis (Supplementary Figures 5B,F). However, mice receiving PPI treatment exhibited no significant differences from the model group in either biochemical markers or pathological scores (Supplementary Figures 5B-G). Taken together, the evidence indicates that PPI exposure does not confer any measurable change in susceptibility to acute pancreatitis in this murine model.

## 4 Discussion

In this study, we combined evidence from a large-scale prospective cohort with complementary animal experiments to investigate the association between regular PPI use and AP risk. While crude analyses suggested a positive association, extensive sensitivity analyses—including stratified analyses, time-varying Cox models, alternative matching methods, and so on—revealed that the results were unstable and largely dependent on analytic choices. Consistent with this, in cerulein-induced pancreatitis models, PPI administration did not alter pancreatic histopathology or inflammatory responses. Together, these findings suggest that both short- and long-term PPI use are unlikely to materially influence the risk of AP or its progression to CP or PC.

No suitable randomized trials have directly addressed this question. Previous studies on PPI pathogenicity have tended to compare PPI to no PPI (Mahmud et al., 2022; Roux et al., 2009) or placebo (Estborn and Joelson, 2015), as performed in subcohort I. However, after choosing an active comparator H_2_RAs to meet the requirement of valid statistical adjustment in subcohort II (Yoshida et al., 2015; Sendor and Stürmer, 2022), our results appeared stratified, as H_2_RAs share similar clinical indications with PPIs and thus reduce confounding by indication. This design minimizes bias from underlying gastrointestinal morbidity and healthcare-seeking behaviors while providing a mechanistically neutral reference group for pancreatic outcomes. These observations highlight the instability of the primary results. In drug-specific analyses, only omeprazole showed associations consistent with the overall PPI effect, echoing signals from a previous case–control study (Blomgren et al., 2002). Nonetheless, methodological and contextual differences—ranging from study design and population characteristics to regional dietary patterns, lifestyle behaviors, and prescribing practices—may account for the divergence from our UK cohort. The persistence of omeprazole signals is more plausibly attributable to non-causal explanations: its widespread use confers longer cumulative exposure and greater statistical power (Torres-Bondia et al., 2022), while channeling toward patients with more severe gastrointestinal comorbidities introduces residual confounding despite PSM and extensive covariate adjustment. Taken together, these findings suggest that the observed association reflects design-related influences rather than a drug-specific effect, underscoring the importance of triangulation with sensitivity analyses and experimental validation. While isolated case reports have described AP as a rare adverse event associated with omeprazole, pantoprazole, and lansoprazole (Youssef et al., 2005; Das et al., 2012; Ocal et al., 2014), and esomeprazole showed null associations consistent with prior evidence (OR: 0.9, 95% CI: 0.2–2.8) (Douros et al., 2013), our comprehensive analyses did not identify any robust effect for these individual PPIs. Taken together, these findings suggest that earlier single-case observations or small-sample signals may reflect idiosyncratic reactions rather than reproducible drug-specific effects. No study has reported a difference in the various types of PPIs on the incidence of AP over 2 years. After correcting for immortal time bias, we replicated our preliminary findings, and the E-value analyses showed that while moderate unmeasured confounding could explain some associations, lower-bound E-values close to 1 suggested limited robustness (VanderWeele and Ding, 2017; Haneuse et al., 2019). Yet HRs and p-values fluctuated widely across various sensitivity analyses, underscoring that extreme estimates most likely reflect sparse events (Greenland et al., 2016) and residual confounding rather than true biological effects, as emphasized in prior methodological work.

As no prior epidemiologic studies have addressed the link between PPI use and chronic pancreatitis, our findings provide the first real-world evidence that long-term PPI therapy does not increase CP risk. In this study, PPI use was not associated with an altered risk of pancreatic cancer when evaluated against H_2_RA use as an active comparator. This finding aligns with large European population-based studies (Hicks et al., 2018; Lassalle et al., 2022), but contrasts with reports from Asian cohorts and a recent meta-analysis that suggested a positive, dose-dependent relationship (Peng et al., 2018; Zhou et al., 2022). We propose that the inconsistencies in previous findings can be attributed to several key factors, including differences in study design (e.g., nested case-control versus prospective cohort), heterogeneity in population genetics and environmental exposures (such as dietary habits and metabolizing phenotypes), and the potential for residual confounding, which underscore the need for more robust evidence. Previous studies showing a stratified truncation effect of PPI administration (Dos Santos et al., 2023), similar to our results, led us to question whether there is indeed a point when PPI causes a quantitative change in the accumulation of the drug, shifting it from a protective to a pathogenic effect. PPI exposure triggers certain autoimmune and immune-mediated inflammatory diseases (Sandholdt et al., 2014; Lin et al., 2021), influences neutrophilic infiltration (H et al., 2011), impairs the gut microbiome (Imhann et al., 2016), and results in permanent dysbiotic disruption (Ortigão et al., 2020).

Our animal experiments were designed to be more robust by ensuring that the long-term PPI dosage in ICR mice is the same as the population dosage and by doubling the short-term dosage to accelerate the cumulative effect of PPIs (Hiromoto et al., 2021; Joo et al., 2013). We used low-dose Cae to create an AP model and to observe the effect of the experimental drug on AP susceptibility (Pan et al., 2017). Ultimately, we did not observe a significant protective or pathogenic effect of regular administration of omeprazole or lansoprazole in the low-dose Cae-induced AP model of ICR mice. Although there are plenty of animal experiments on PPIs for the treatment of AP that have observed a significant anti-inflammatory effect with PPIs (Hackert et al., 2010), few previous studies have addressed the relationship between PPI administration and AP. In a study by Cai et al. (2007), omeprazole, at different concentrations or with different action times, had no direct inhibitory effect on the exocrine activity of isolated pancreatic cells. Burdan et al. (2000) performed a rat test with short-term (3 days) and long-term (6 weeks) intraperitoneal administration of omeprazole, in line with our experimental results.

In this study, the clinical data analysis and animal experiments showed great heterogeneity and contradictory results, which is common in the exploration of new research orientations. Despite extensive adjustments, causality could not be directly established. First, dose-effect relationships are an unavoidable consideration in the study of drug-disease relationships. We could not complete a dose-effect analysis because participants were not asked to include specific drug doses in their questionnaires. However, the data are credible and can be utilized for research (He et al., 2021). Second, covariates such as smoking and alcohol use reflected short-term rather than cumulative exposures, potentially distorting associations; short-term alcohol misuse is a stronger predictor of AP onset. Indeed, our E-value analyses demonstrated that unmeasured confounding—including indication for use and socioeconomic factors—may bias results, and the potential for confounding by indication and healthy volunteer bias in UK Biobank further limits generalizability. Third, the lack of sufficient representativeness of the UK Biobank population, which is a cohort of mainly middle-aged and older Caucasians, may limit the generalizability of these findings to the general population. Animal experiments have certain limitations. ICR mice cannot completely mimic humans, and the actual effect of a drug is objectively affected by various factors such as the manufacturer, formulation, batches, administration method, and experimental environment. However, it is easier to obtain pancreatic tissues for pathological evaluation in mice, which is the gold standard for the diagnosis of AP.

In summary, the results of this study suggest that participants regularly treated with PPIs have no increased or decreased risk of pancreatic diseases compared to non-PPI participants or participants treated with H_2_RAs. Researchers should conduct multiple avenues of validation and be more prudent in presenting results when they obtain positive results from data analysis to avoid misinformation. Further prospective randomized controlled clinical studies are recommended to obtain high-quality data.

## Data Availability

The datasets presented in this study can be found in online repositories. The names of the repository/repositories and accession number(s) can be found below: https://www.ukbiobank.ac.uk/.
