# Supplementary material for: Proton pump inhibitor use and pancreatic risk: evidence from the UK biobank participants and animal experiments
Source: Front Pharmacol. 2025 Oct 8;16:1673200. doi: 10.3389/fphar.2025.1673200 (PMC12540142; doi:10.3389/fphar.2025.1673200)
Supplement: Supplementary file 1 [file Supplementaryfile1.pdf]

Proton pump inhibitor use and pancreatic risk: evidence from the UK Biobank participants and animal experiments

Supplementary Materials

Xin Gao [1,2,i], Zouhua Xu [3,4,i], Qingxie Liu [1,2,i], Chenchen Yuan[1,2], Xiaowu Dong [1,2], Xiaolei Shi [1,2], Qingtian Zhu [1,2], Keyan Wu [1,2], Hongwei Xu [3,4], Jiajia Pan [1,2], Guotao Lu [1,2], Weiming Xiao [1,2]*, Shengfeng Wang [5,6]*, Yaodong Wang [3,4]*

Supplementary Text Material 1 Covariates involved in analysis with chronic pancreatitis and pancreatic cancer endpoints 2
Supplementary Table 1 Detailed codes for drugs involved in this study 3
Supplementary Table 2 Detailed codes and classification of the main outcome events 7
Supplementary Table 3 Distribution and characteristics of participants with PPI vs H_2RA use, along with those with no regular prescriptions in the UK Biobank 8
Supplementary Table 4 Results of univariate Cox regression of covariates with AP outcome in two cohorts 9
Supplementary Table 5 Sensitivity analysis A: Stratified and interaction analyses with key variables with the onset of AP 10
Supplementary Table 6 Sensitivity analysis B: results of E-value analyses 12
Supplementary Table 7 Sensitivity analysis D: results after correcting for immortal time bias 14
Supplementary Table 8 Sensitivity analysis D: results of multivariate Cox regression analysis of the whole population cohort 14
Supplementary Table 9 Sensitivity analysis g: Firth's penalized partial likelihood correction results after limiting the number of follow-up visits 15
Supplementary Table 10 Sensitivity analysis h: Alternative matching methods 15
Supplement Fig. 2 Standardized mean differences (SMD) of different cohorts 17
Supplement Fig. 3 Comparison of cumulative probability and the cumulative number of events of chronic pancreatitis and pancreatic cancer between subcohort I and subcohort II 18
Supplement Fig. 4 Crude and adjusted hazard ratios (HRs) for the association between pancreatic disorders and regular PPI use in two cohorts 19
Supplement Fig. 5 Flowchart and experimental results of the comparison between PPI adoption and low-dose Cae-induced AP in ICR mice 20

Supplementary Text Material 1 Covariates involved in analysis with chronic pancreatitis and pancreatic cancer endpoints

In the cohort analysis of participants who had chronic pancreatitis, sociodemographic factors (sex, age, ethnicity, Townsend deprivation index), lifestyle factors (body mass index (BMI), smoking status, alcohol intake frequency), diagnosis of diabetes, disorders of lipoprotein metabolism and other lipidemias, cholelithiasis, and history of acute pancreatitis were covariates.

In the cohort analysis with an ending of pancreatic cancer, sociodemographic factors (sex, age, ethnicity, Townsend deprivation index), lifestyle factors (body mass index (BMI), smoking status, alcohol intake frequency), diagnosis of diabetes, disorders of lipoprotein metabolism and other lipidemias, cholelithiasis, history of acute pancreatitis, and chronic pancreatitis were covariates.

Supplementary Table 1 Detailed codes for drugs involved in this study

Variable type	Code
PPI	
Omeprazole	data-coding 100628(coding 5); data-coding 4(coding 1140865634, 1140909578, 1141187060, 1141190552)
Rabeprazole	data-coding 4(1140850836, 1140850960, 1141168584, 1141168590)
Esomeprazole	data-coding 4(1141177526, 1141177532)
Lansoprazole	data-coding 4(1140864752, 1141168822, 1141180462, 1141184174, 1140923688)
Pantoprazole	data-coding 4(1141164616, 1140929012)
H_2RA	
Ranitidine	data-coding 100628(coding 4); data-coding 4(1140879406, 1140916980,1140922658, 1140922662, 1141157018, 1141166508, 1141167418, 1141173202, 1141184376, 1141188426)
Cimetidine	data-coding 4(1140865426, 1140865492, 1140865496, 1140865510, 1140865512, 1140865524, 1140865530, 1140879402, 1140909500, 1140921954)
Famotidine	data-coding 4(1140865608, 1140881504, 1140909496, 1141193276, 1141199916, 1141200376, 1141200442, 1141200652, 1141200654)
Nizatidine	data-coding 4(1140865618, 1140865624)
Other covariate drugs[1]	
5-aminosalicylic acid (mesalamine)	data-coding 4(1140865578, 1140865580, 1140865588, 1140865598, 1140910598, 1141152746, 1141153242, 1141153248, 1141162746)
Mercaptopurine (6-MP)	data-coding 4(1140869842, 1140869844, 1140910596)

Acetaminophen	data-coding 4(1140850904, 1140855808, 1140856234, 1140856238, 1140856240, 1140856242, 1140856244, 1140856246, 1140856248, 1140856340, 1140856342, 1140856346, 1140856348, 1140856392, 1140856398, 1140856408, 1140856416, 1140856418, 1140856420, 1140856428, 1140856430, 1140856432, 1140856434, 1140856436, 1140856438, 1140856440, 1140856442, 1140861804, 1140863000, 1140863002, 1140863514, 1140868240, 1140868266, 1140868268, 1140868274, 1140868278, 1140868280, 1140868286, 1140868302, 1140868304, 1140868306, 1140868312, 1140868322, 1140868324, 1140868326, 1140868328, 1140871920, 1140871924, 1140871926, 1140871968, 1140872036, 1140872044, 1140882218, 1140882220, 1140882222, 1140882248, 1140882258, 1140882262, 1140882266, 1140882394, 1140882396, 1140884404, 1140884408, 1140888772, 1140909474, 1140911096, 1140911758, 1140923346, 1140923348, 1140923350, 1140925778, 1140926016, 1140926020, 1140927318, 1140928260, 1140928946, 1140928954, 1140928956, 1141156856, 1141163764, 1141168122, 1141168560, 1141168562, 1141168646, 1141168648, 1141168650, 1141169026, 1141169210, 1141169692, 1141169808, 1141172966, 1141178052, 1141182532, 1141182534, 1141183332, 1141184186, 1141184226, 1141185058, 1141185060, 1141188500, 1141188512, 1141188516, 1141188780, 1141188784, 1141189008, 1141189010, 1141190006, 1141190956, 1141200736, 1141201642, 2038460150)
Azathioprine	data-coding 4(1140869930, 1140869932, 1140869940, 1140909864, 1141145996)
Codeine	data-coding 4(1140855740, 1140856336, 1140856394, 1140856396, 1140856406, 1140856422, 1140856426, 1140856454, 1140856456, 1140856458, 1140862988, 1140862994, 1140865652, 1140865654, 1140871682, 1140871684, 1140871686, 1140871688, 1140882268, 1140882392, 1140884444, 1140884452, 1140884464, 1140888706,

	1140921988, 1140923344, 1140927384, 1141168554, 1140856340, 1140856348, 1140856416, 1140856430, 1140856434, 1140856440, 1140856442, 1140861804, 1140863514, 1140868286, 1140871920, 1140882394, 1140882396, 1140923346, 1140923350, 1140927318, 1140928260, 1141168122, 1141168648, 1141168650, 1141169692, 1141169808, 1141178052, 1141189008, 1141189010, 1141190006)
Erythromycin	data-coding 4(1140873608, 1140873618, 1140873620, 1140873622, 1140873630, 1140873632, 1140873672, 1140873674, 1140873676, 1140873678, 1140873680, 1140873682, 1140873690, 1140873692, 1140873694, 1140873696, 1140873698, 1140873700, 1140873702, 1140873708, 1140873712, 1140873714, 1140873718, 1140873722, 1140882356, 1140882358, 1140882360, 1140884008, 1140884012, 1140884016, 1140928674, 1141163532, 1141163716, 1141163778, 1141163784, 1141171614, 1141195068)
Interferon alpha	data-coding 4(1140864560, 1140864562, 1140870112, 1140870114, 1140870116, 1140870118, 1140870120, 1140870122, 1140870124, 1140888636, 1140888638, 1140888640, 1140916886, 1140916888, 1140922902, 1140922906, 1140922910, 1140922914, 1140923632, 1141146368, 1141146370, 1141146372, 1141146374, 1141146488, 1141150960, 1141162690, 1141162692, 1141162694, 1141162752, 1141166864, 1141169674, 1141171732, 1141171736, 1141171740, 1141171744, 1141171746, 1141172484, 1141181502, 1141181630, 1141186792, 1141186794, 1141186796, 1141186800, 1141186802)
Isoniazid	data-coding 4(1140857158, 1140857216, 1140873918, 1140873920, 1140873986, 1140874096, 1140882372, 1140882548, 1140882550, 1140910506, 1140910508)
L-asparaginase	-
Lisinopril	data-coding 4(1140860714, 1140860738, 1140864910, 1140864952, 1141200726)
Methimazole	-

Methylprednisolone	data-coding 4(1140874976, 1140883026)
Metronidazole	data-coding 4(1140857232, 1140857234, 1140874014, 1140874030, 1140874032, 1140874034, 1140874036, 1140874038, 1140874040, 1140874042, 1140874044, 1140874046, 1140874048, 1140874114, 1140888624, 1141157364)
Prednisone	data-coding 4(1140857612, 1140857614, 1140857672, 1140865832, 1140865838, 1140865840, 1140868364, 1140874930, 1140874936, 1140874940, 1140874944, 1140874950, 1141157402)
Simvastatin	data-coding 4(1140861958, 1140881748, 1141200040)
Sorafenib	-
Sulindac	data-coding 4(1140871604, 1140871606)
Tamoxifen	data-coding 4(1140858348, 1140870164, 1140870170, 1140870176, 1140870182, 1141170264)
Tetracycline	data-coding 4(1140857044, 1140857050, 1140857052, 1140857060, 1140873364, 1140873366, 1140873368, 1140873370, 1140873372, 1140873374, 1140873376, 1140873384, 1140873450, 1140873456, 1140873458, 1140873460, 1140873462, 1140873464, 1140873466, 1140873476)
Trimethoprim-sulfamethoxazole	data-coding 4(1140873780, 1140873942, 1140873944, 1140873946, 1140873948)
Valproic acid	data-coding 4(1140872198, 1140872200, 1140872214, 1140872216, 1141182592)

1: Cimetidine was not repeatedly included in the covariate group.

Supplementary Table 2 Detailed codes and classification of the main outcome events

Acute pancreatitis	ICD-10: K85, K85.0, K85.1, K85.2, K85.3, K85.8, K85.9;<br>ICD-9: 5770
Chronic pancreatitis	ICD-10: K86.0, K86.1;<br>ICD-9: 5771
Pancreatic cancer	ICD-10: C25, C25.0, C25.1, C25.2, C25.3, C25.4, C25.7, C25.8, C25.9

Supplementary Table 3 Distribution and characteristics of participants with PPI vs H_2RA use, along with those with no regular prescriptions in the UK Biobank

Characteristics	regular PPI users (N=54 453)	regular H_2RA users (N=8 293)	Blank control users (N=426 648)	Total (N=489 394)	*p overall*
Age, median (IQR)	62.0 (56.0,67.0)	60.0 (52.0,65.0)	58.0 (50.0,63.0)	58.0 (51.0,64)	<0.001
Sex					0.609
Female	29772 (54.7)	4544 (54.8)	232450 (54.5)	266766 (54.5)	
Male	24681 (45.3)	3749 (45.2)	194198 (45.5)	222628 (45.5)	
Race					<0.001
White	51935 (95.4)	7857 (94.7)	402463 (94.3)	462255 (94.5)	
other	2518 (4.62)	436 (5.26)	24185 (5.67)	27139(5.5)	
Index of multiple deprivation, median (IQR)	-1.94 (-3.55,1.02)	-1.76 (-3.43,1.32)	-2.19 (-3.67,0.42)	-2.16 (-3.65,0.50)	<0.001
BMI, median (IQR)	28.3 (25.6,31.7)	28.0 (25.3,31.4)	26.5 (24.0,29.6)	26.7 (24.1,29.9)	<0.001
Alcohol intake frequency					<0.001
never	6342 (11.6)	858 (10.3)	31721 (7.43)	38921 (8.0)	
≤3 times a month	14512 (26.7)	2043 (24.6)	94576 (22.2)	111131 (22.7)	
1-4 times a week	23893 (43.9)	3690 (44.5)	212319 (49.8)	239902 (49.0)	
Daily or almost daily	9706 (17.8)	1702 (20.5)	88032 (20.6)	99440 (20.3)	
Smoking status					<0.001
Never	26134 (48.0)	3798 (45.8)	238675 (55.9)	268607 (54.9)	
Previous	22925 (42.1)	3331 (40.2)	143456 (33.6)	169712 (34.7)	
Current	5394 (9.91)	1164 (14.0)	44517 (10.4)	51075 (10.4)	
Diabetes	5478 (10.1)	672 (8.10)	19449 (4.56)	25599 (5.2)	<0.001
Hyperlipidemia	10108 (18.6)	1211 (14.6)	49928 (11.7)	61247 (12.5)	<0.001
Cholelithiasis	3113 (5.72)	445 (5.37)	12700 (2.98)	16258 (3.3)	<0.001
Short-term therapeutic endoscopic retrograde operations	439 (0.81)	66 (0.80)	1578 (0.37)	2083 (0.4)	<0.001
types of relative drugs					<0.001
0	27831 (51.1)	4371 (52.7)	305454 (71.6)	337656 (69.0)	
1	17335 (31.8)	2884 (34.8)	101315 (23.7)	121534 (24.8)	
2	7416 (13.6)	851 (10.3)	17234 (4.04)	25501 (5.2)	
3	1735 (3.19)	181 (2.18)	2542 (0.60)	4458 (0.9)	
4	125 (0.23)	6 (0.07)	96 (0.02)	227 (0.0)	
5	8 (0.01)	0 (0.00)	6 (0.00)	14 (0.0)	
6	3 (0.01)	0 (0.00)	1 (0.00)	4 (0.0)	

Categorical variables are described as percentages and compared using the chi-square test or Fisher's exact test. Continuous variables are described as medians and interquartile ranges (IQR) and were compared using the Wilcoxon rank-sum test.
BMI: body mass index

Supplementary Table 4 Results of univariate Cox regression of covariates with AP outcome in two cohorts

**Acute Pancreatitis**	**PPI *vs* non-PPI subcohort**		**PPI *vs* H_2RA subcohort**	
	**HR (95% CI)**	***P***	**HR(95% CI)**	***P***
Age	1.04 (1.03,1.04)	< 0.001	1.01 (1.00,1.02)	0.02
Sex (Ref.=female)		< 0.001		0.002
male	1.16 (1.07,1.25)		1.26 (1.09,1.46)	
Race (Ref.=other)		0.08		0.4
White	1.17 (0.98,1.40)		1.17 (0.81,1.70)	
Index of multiple deprivation	1.06 (1.05,1.07)	< 0.001	1.05 (1.03,1.07)	< 0.001
BMI	1.08 (1.07,1.09)	< 0.001	1.05 (1.04,1.06)	< 0.001
Alcohol intake frequency		< 0.001		0.06
never	Ref		Ref	
≤3 times a month	0.83 (0.72,0.94)		0.90 (0.70,1.14)	0.37
1-4 times a week	0.61 (0.53,0.69)		0.76 (0.60,0.95)	0.02
Daily or almost daily	0.62 (0.54,0.71)		0.78 (0.59,1.02)	0.07
Smoking status		< 0.001		0.004
Never	Ref		Ref	
Previous	1.34 (1.23,1.45)		1.24 (1.06,1.46)	0.008
Current	1.50 (1.34,1.69)		1.41 (1.11,1.78)	0.005
Diabetes	1.89 (1.65,2.16)	< 0.001	1.60 (1.29,1.98)	< 0.001
Hyperlipidemia	0.97 (0.87,1.09)	0.6	0.70 (0.57,0.87)	< 0.001
Cholelithiasis	4.66 (4.18,5.2)	< 0.001	2.35 (1.89,2.93)	< 0.001
Short-term therapeutic endoscopic retrograde operations	7.57 (6.02,9.53)	< 0.001	4.04 (2.64,6.17)	< 0.001
Types of relative drugs		< 0.001		< 0.001
0	Ref		Ref	
1-3 types	1.62 (1.50,1.75)		1.42 (1.22,1.66)	< 0.001
≥4 types	4.09 (1.53,10.91)		2.47 (0.79,7.69)	0.12

Supplementary Table 5 Sensitivity analysis A: Stratified and interaction analyses with key variables with the onset of AP

Table a: Stratified Analysis by PPI Type in PPI *vs* non-PPI subcohort

PPI *vs* non-PPI subcohort	Events	Person-years	Crude incidence rate (95% CI)	Crude HR		PSM-adjusted HR	
				HR (95% CI)	*p*	HR (95% CI)	*p*
**PPI**	617	94.64	2.63 (2.40,2.88)	2.66 (2.43,2.91)	< 0.001	1.95 (1.71,2.22)	< 0.001
**Omeprazole**	399	90.45	2.36 (2.12,2.63)	2.39 (2.15,2.65)	<0.001	1.44 (1.27,1.64)	<0.001
**Rabeprazole**	18	128.58	3.09 (1.83,4.89)	3.10 (1.95,4.93)	<0.001	1.81 (1.13,2.88)	0.0131
**Esomeprazole**	24	77.41	1.86 (1.19,2.77)	1.87 (1.25,2.79)	0.002	1.08 (0.72,1.61)	0.71
**Lansoprazole**	214	94.39	2.36 (2.05,2.72)	2.38 (2.07,2.74)	<0.001	1.40 (1.21,1.63)	<0.001
**Pantoprazole**	18	142.21	3.42 (2.02,5.40)	3.43 (2.16,5.46)	<0.001	2.00 (1.25,3.19)	0.004

Table b: Stratified Analysis by PPI Type in PPI vs H_2RA subcohort

PPI *vs* H_2RA subcohort	Crude incidence rate (95% CI)	Crude HR				PSM-adjusted HR			
		Administration time <2 years		Administration time ≥2 years		Administration time <2 years		Administration time ≥2 years	
		HR (95% CI)	*p*	HR (95% CI)	*p*	HR (95% CI)	*p*	HR (95% CI)	*p*
**PPI**	1.35(1.06,1.75)	0.38(0.22,0.64)	< 0.001	1.89 (1.12,3.2)	0.02	0.49 (0.25,0.99)	0.047	1.79 (1.29,2.48)	< 0.001
**Omeprazole**	0.98(0.84,1.14)	0.72 (0.49,1.05)	0.09	0.99 (0.84,1.17)	0.9	0.43(0.20,0.92)	0.03	1.50(1.10,2.06)	0.01
**Rabeprazole**	1.42(0.84,2.26)	1.88 (0.26,13.52)	0.53	1.57 (0.97,2.54)	0.07	0	1	2.41(0.89,6.51)	0.08
**Esomeprazole**	0.84(0.54,1.27)	1.27 (0.47,3.44)	0.64	0.82 (0.53,1.28)	0.39	0	1	1.39(0.57,3.38)	0.47
**Lansoprazole**	1.05(0.89,1.24)	0.83 (0.55,1.26)	0.37	1.07 (0.90,1.27)	0.47	1.11(0.48,2.56)	1.43	1.16(0.78,1.73)	0.46
**Pantoprazole**	1.57(0.93,2.51)	2.15 (0.68,6.77)	0.19	1.54 (0.92,2.57)	0.10	4.17(0.57,30.77)	0.16	2.01(0.64,6.3)	0.23

Table c: Stratified and Interaction Analyses of Key Variables in unmatched cohort

Variable	Subcohort I: PPI *vs* non-PPI cohort			Subcohort II: PPI *vs* H_2RA cohort					
	Unmatched cohort			Unmatched cohort					
				Administration time ＜2 years			Administration time ≥2 years		
	HR (95% CI)	P value	P for interaction	HR (95% CI)	P value	P for interaction	HR (95% CI)	P value	P for interaction
**sex**			0.13			0.1			0.77
**female**	2.48 (2.19, 2.82)	<0.001		0.26 (0.13, 0.5)	<0.001		1.42 (0.97, 2.09)	0.07	
**male**	2.86 (2.52, 3.25)	<0.001		0.65 (0.26, 1.63)	0.35		1.54 (1.05, 2.28)	0.03	
**age**			0.004			0.62			0.03
**≤50**	3.61 (2.74, 4.75)	<0.001		0.27 (0.03, 2.35)	0.23		1.07 (0.59, 1.93)	0.82	
**50–67**	2.47 (2.21, 2.76)	<0.001		0.44 (0.23, 0.84)	0.01		1.33 (0.95, 1.85)	0.09	
**>67**	2.04 (1.69, 2.46)	<0.001		0.32 (0.11, 0.91)	0.03		3.99 (1.48, 10.8)	0.01	
**Races**			0.68			0.03			0.98
**White**	2.65 (2.41, 2.9)	<0.001		0.4 (0.23, 0.69)	0.001		1.41 (1.07, 1.85)	0.02	
**other**	2.89 (1.9, 4.4)	<0.001		0.01 (0, 0.16)	0.001		—	—	
**BMI**			0.001			0.77			0.96
**<24**	3.82 (2.91, 5)	<0.001		0.27 (0.07, 0.07)	0.06		1.46 (0.67, 3.21)	0.34	
**24-28**	2.4 (2, 2.86)	<0.001		0.31 (0.11, 0.89)	0.03		1.57 (0.88, 2.77)	0.13	
**≥28**	2.13 (1.9, 2.39)	<0.001		0.44 (0.22, 0.9)	0.02		1.41 (1.01, 1.98)	0.045	
**Index of multiple deprivation**			0.6			0.67			0.73
**Low**	2.88 (2.35, 3.52)	<0.001		0.51 (0.12, 2.23)	0.37		1.74 (0.88, 3.44)	0.11	
**Medium**	2.55 (2.26, 2.88)	<0.001		0.48 (0.21, 1.06)	0.07		1.35 (0.94, 1.93)	0.11	
**High**	2.56 (2.15, 3.05)	<0.001		0.3 (0.13, 0.68)	0.004		1.64 (0.96, 2.79)	0.07	
**Other drug type**			0.97			0.75			0.9
**0**	2.47 (2.16, 2.83)	<0.001		0.37 (0.15, 0.88)	0.03		1.4 (0.93, 2.11)	0.1	
**1-3 types**	2.41 (2.12, 2.73)	<0.001		0.42 (0.22, 0.83)	0.01		1.51 (1.04, 2.18)	0.03	
**≥4 types**	2.47 (0.26, 23.78)	0.43		—	—		—	—	

Supplementary Table 6 Sensitivity analysis B: results of E-value analyses

Table a: PPI *vs* non-PPI subcohort

PPI *vs* non-PPI subcohort	Crude analysis			PS match analysis		
	HR (95% CI)	E-value (point estimate)	E-value (confidence interval)	HR (95% CI)	E-value (point estimate)	E-value (confidence interval)
**PPI**	2.66 (2.43,2.91)	4.76	4.29	1.95 (1.71,2.22)	3.31	2.81
**Omeprazole**	2.39 (2.15,2.65)	4.21	3.72	1.44 (1.27,1.64)	2.24	1.86
**Rabeprazole**	3.10 (1.95,4.93)	5.65	3.31	1.81 (1.13,2.88)	3.02	1.51
**Esomeprazole**	1.87 (1.25,2.79)	3.15	1.81	1.08 (0.72,1.61)	1.37	1
**Lansoprazole**	2.38 (2.07,2.74)	4.19	3.56	1.40 (1.21,1.63)	2.15	1.71
**Pantoprazole**	3.43 (2.16,5.46)	6.32	3.74	2.00 (1.25,3.19)	3.41	1.81

Table b: Crude analysis in PPI *vs* H_2RA subcohort

PPI *vs* H_2RA subcohort	Crude analysis					
	Administration time < 2 years			Administration time ≥ 2 years		
	HR (95% CI)	E-value (point estimate)	E-value (confidence interval)	HR (95% CI)	E-value (point estimate)	E-value (confidence interval)
**PPI**	0.38(0.22,0.64)	4.70	2.50	1.89 (1.12,3.2)	3.19	1.49
**Omeprazole**	0.72 (0.49,1.05)	2.12	1	0.99 (0.84,1.17)	1.11	1
**Rabeprazole**	1.88 (0.26,13.52)	3.17	1	1.57 (0.97,2.54)	2.52	1
**Esomeprazole**	1.27 (0.47,3.44)	1.86	1	0.82 (0.53,1.28)	1.74	1
**Lansoprazole**	0.83 (0.55,1.26)	1.71	1	1.07 (0.90,1.27)	1.34	1
**Pantoprazole**	2.15 (0.68,6.77)	1	1	1.54 (0.92,2.57)	2.45	1

Table c: PSM analysis in PPI *vs* H_2RA subcohort

PPI *vs* H_2RA subcohort	PS match analysis					
	Administration time < 2 years			Administration time ≥ 2 years		
	HR (95% CI)	E-value (point estimate)	E-value (confidence interval)	HR (95% CI)	E-value (point estimate)	E-value (confidence interval)
**PPI**	0.49 (0.25,0.99)	3.5	1.11	1.79 (1.29,2.48)	2.98	1.9
**Omeprazole**	0.43(0.20,0.92)	4.08	1.39	1.50(1.10,2.06)	2.37	1.43
**Rabeprazole**	0	-	-	2.41(0.89,6.51)	2.21	1

**Esomeprazole**	0	-	-	1.39(0.57,3.38)	2.13	1
**Lansoprazole**	1.11(0.48,2.56)	1.46	1	1.16(0.78,1.73)	1.59	1
**Pantoprazole**	4.17(0.57,30.77)	7.81	1	2.01(0.64,6.3)	3.43	1

Supplementary Table 7 Sensitivity analysis D: results after correcting for immortal time bias

PS matched analysis	PPI *vs* non-PPI subcohort		PPI *vs* H_2RA subcohort			
	HR (95% CI)	*P*	Administration time < 2 years		Administration time ≥ 2 years	
			HR (95% CI)	*P*	HR (95% CI)	*P*
**PPI**	1.76(1.55,2.00)	< 0.001	0.49(0.24,0.99)	0.05	1.79(1.29,2.48)	< 0.001
**Omeprazole**	1.40(1.23,1.59)	< 0.001	0.43(0.20,0.91)	0.03	1.50(1.10,2.06)	0.01
**Rabeprazole**	1.77(1.11,2.83)	0.02	-	-	2.43(0.90,6.56)	0.08
**Esomeprazole**	1.06(0.71,1.59)	0.78	0	1	1.39(0.57,3.38)	0.47
**Lansoprazole**	1.37(1.18,1.59)	< 0.001	1.11(0.48,2.56)	0.81	1.16(0.78,1.73)	0.46
**Pantoprazole**	1.96(1.23,3.12)	0.005	4.18(0.57,30.86)	0.16	2.01(0.64,6.3)	0.23

Supplementary Table 8 Sensitivity analysis D: results of multivariate Cox regression analysis of the whole population cohort

	PPI *vs* non-PPI whole population cohort		PPI *vs* H_2RA whole population cohort			
	HR (95% CI)	*P*	Administration time < 2 years		Administration time ≥ 2 years	
			HR (95% CI)	*P*	HR (95% CI)	*P*
**PPI**	1.83 (1.67,2.01)	< 0.001	0.52 (0.30,0.91)	0.02	1.42 (1.07,1.87)	0.01
**Omeprazole**	1.68 (1.50,1.87)	<0.001	0.74 (0.50,1.10)	0.14	1.02 (0.86,1.20)	0.84
**Rabeprazole**	2.17 (1.37,3.46)	0.001	0.90 (0.12,7.00)	0.92	1.56 (0.96,2.53)	0.07
**Esomeprazole**	1.31 (0.88,1.96)	0.19	1.11 (0.40,3.12)	0.84	0.84 (0.54,1.31)	0.43
**Lansoprazole**	1.59 (1.38,1.84)	<0.001	0.93 (0.61,1.43)	0.76	1.02 (0.86,1.22)	0.81
**Pantoprazole**	2.28 (1.43,3.63)	<0.001	2.02 (0.63,6.50)	0.24	1.49 (0.89,2.49)	0.13

Supplementary Table 9 Sensitivity analysis g: Firth's penalized partial likelihood correction results after limiting the number of follow-up visits

	Subcohort I: PPI *vs* non-PPI cohort		Subcohort II: PPI *vs* H_2RA cohort		
	**Crude HR (95% CI)**	**PSM-adjusted HR (95% CI)**	**Administration time**	**Crude HR (95% CI)**	**PSM-adjusted HR (95% CI)**
**Follow-up times ≥ 2**	1.06 (0.74, 1.49)	349.47 (21.09, 5791.2)	＜2 years	0.26 (0.06, 2.37)	—
			⩾2 years	3.30 (0.89, 29.18)	5.73 (1.32, 53.60)
**Follow-up times ≥ 3**	1.07 (0.41, 2.35)	64.50 (3.07, 1353.48)	＜2 years	—	—
			⩾2 years	—	2.68 (0.14, 391.68)
**Follow-up times ≥ 4**	1.23 (0.01, 15.12)	—		—	

Hazard ratios (HRs) and 95% confidence intervals (95% CIs) relate to Firth's bias correction.

Supplementary Table 10 Sensitivity analysis h: Alternative matching methods

**Methods**	Subcohort I: PPI *vs* non-PPI cohort		Subcohort II: PPI *vs* H_2RA cohort			
	**After matched cohort**		**After matched cohort**			
			**Administration time ＜2 years**		**Administration time ≥2 years**	
	**HR (95% CI)**	**P value**	**HR (95% CI)**	**P value**	**HR (95% CI)**	**P value**
**DRS 1:1 matching**	1.77 (1.55, 2.01)	< 0.001	0.42 (0.15, 1.18)	0.1	1.87 (1.35, 2.58)	< 0.001
**entropy balancing matching**	1.86 (1.69, 2.04)	< 0.001	0.40 (0.23, 0.70)	0.007	1.52 (1.14, 2.01)	0.004

Supplement Fig. 1 Cohorts of patients with or without regular use of PPI or H_2RA

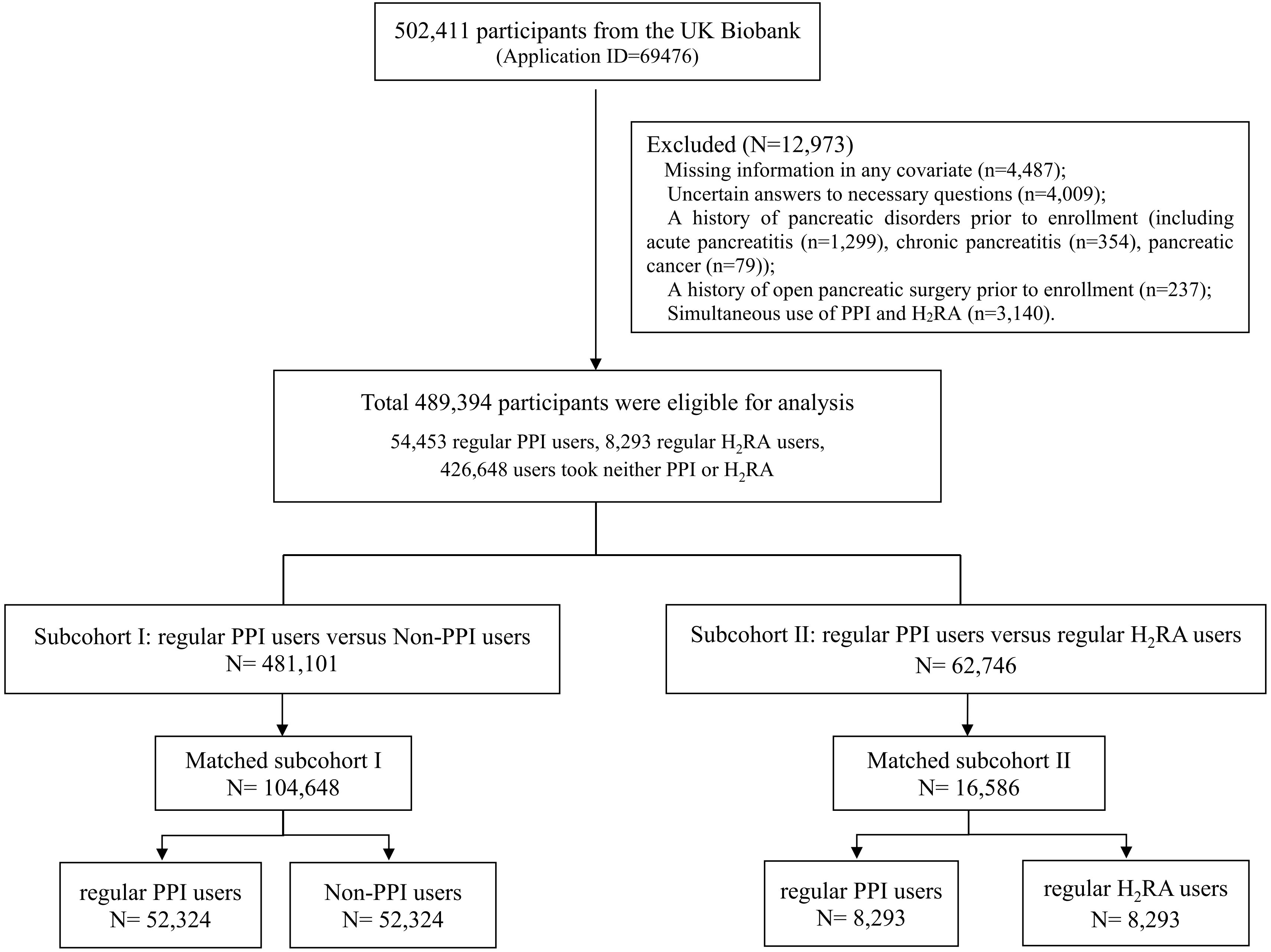

Supplement Fig. 2 Standardized mean differences (SMD) of different cohorts

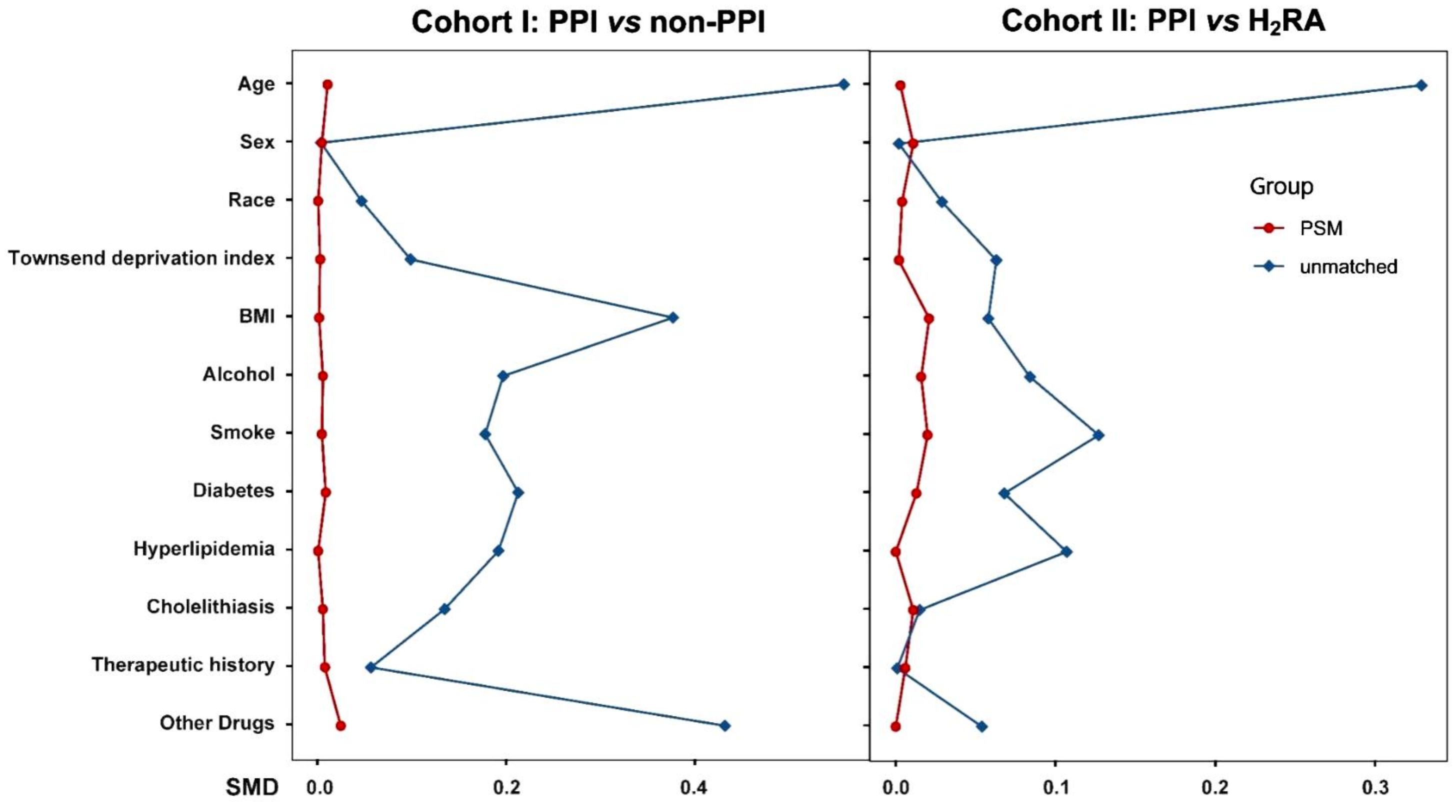

Supplement Fig. 3 Comparison of cumulative probability and the cumulative number of events of chronic pancreatitis and pancreatic cancer between subcohort I and subcohort II

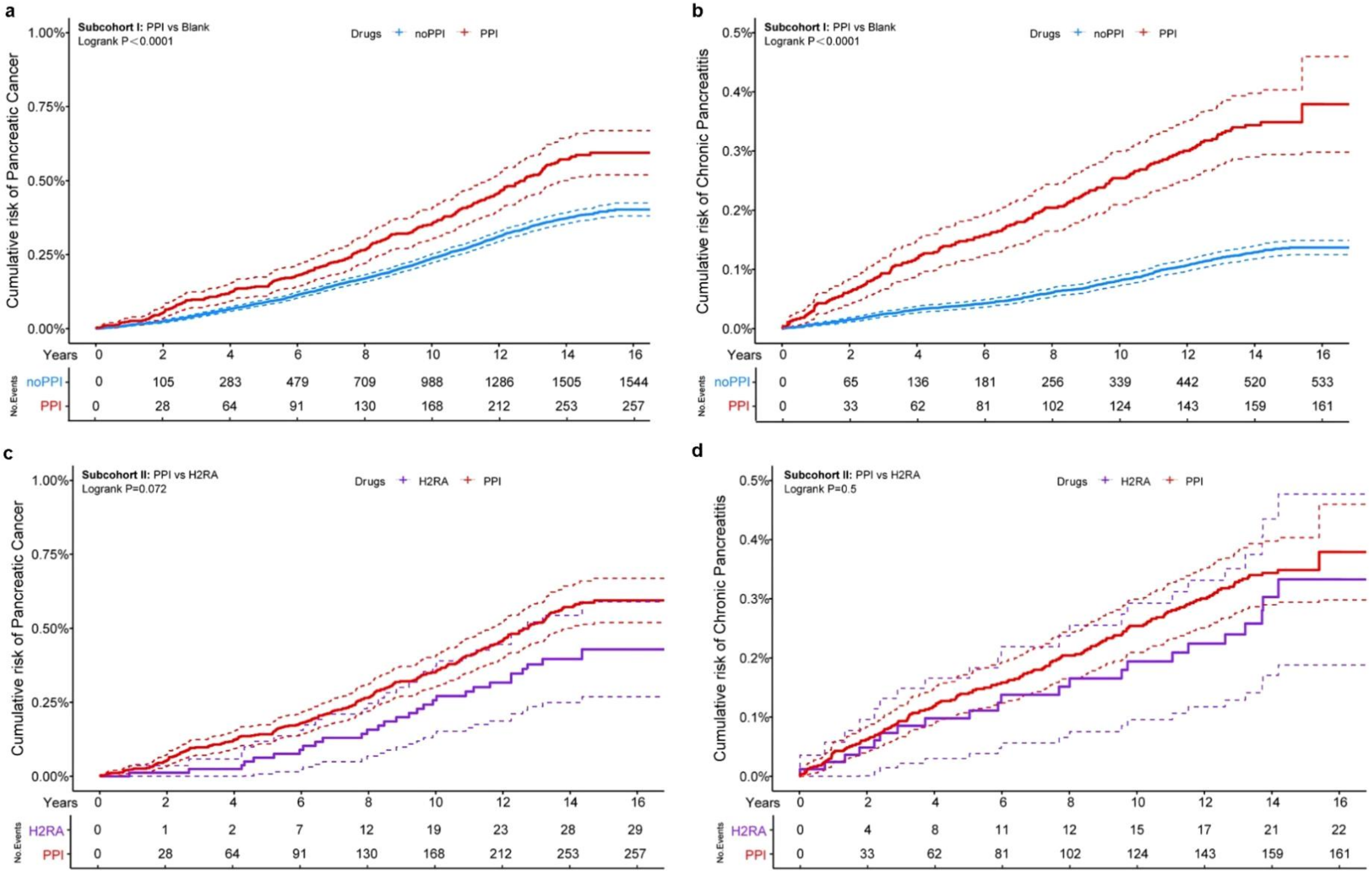

Supplement Fig. 4 Crude and adjusted hazard ratios (HRs) for the association between pancreatic disorders and regular PPI use in two cohorts

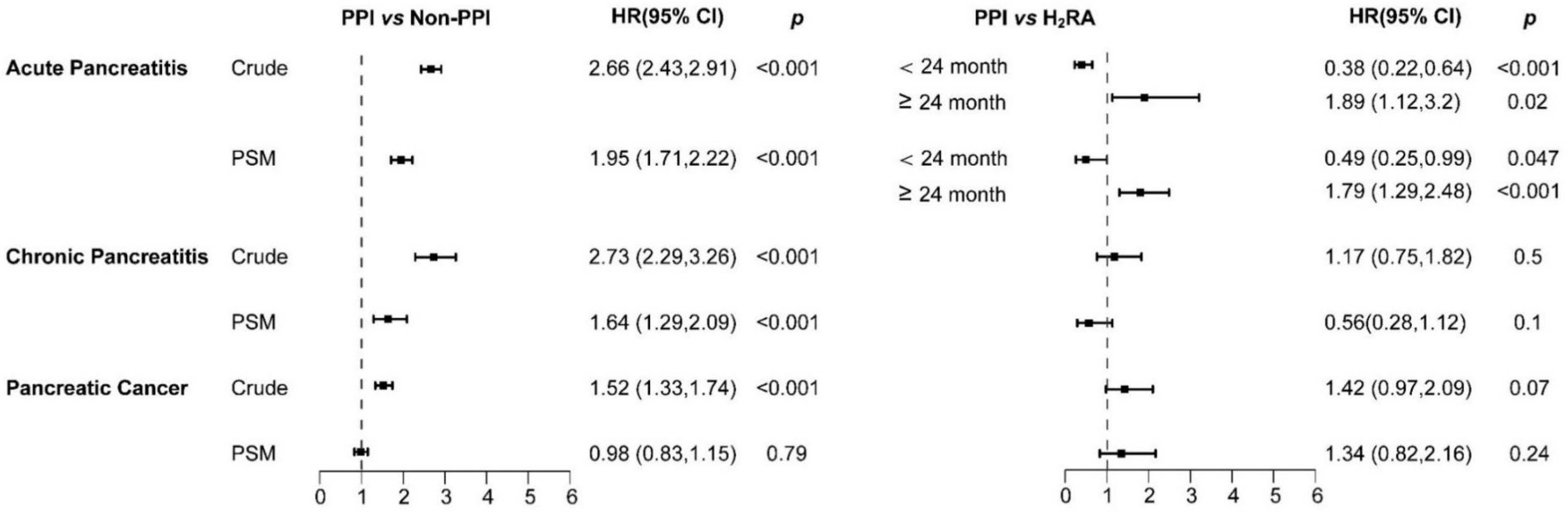

Supplement Fig. 5 Flowchart and experimental results of the comparison between PPI adoption and low-dose Cae-induced AP in ICR mice

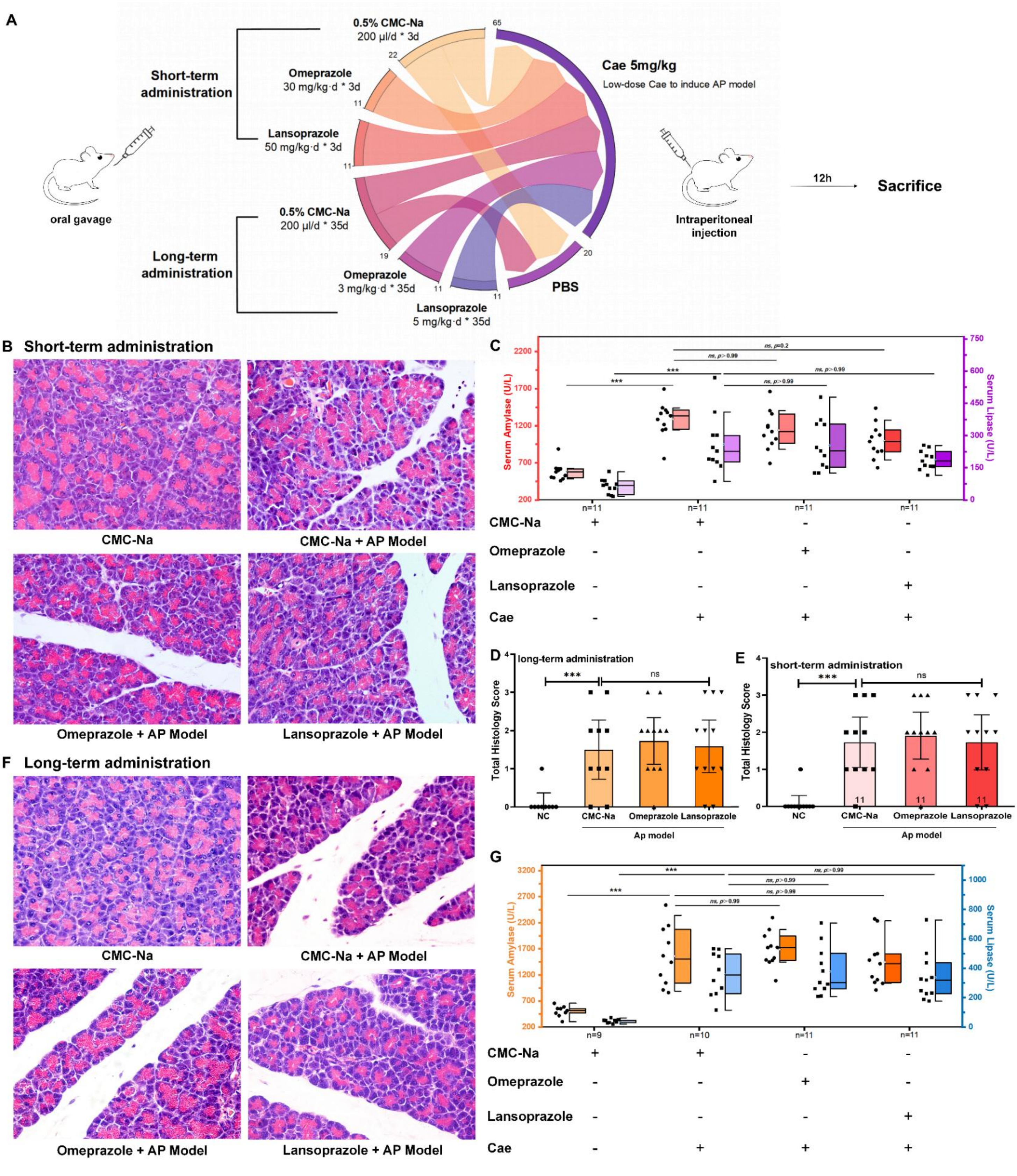

The grouping of all experimental mice, mode of administration, drug concentration, and modeling process are presented in a visual flowchart (a). ICR mice in the short-term gavage groups were assessed by group pathology (b), amylase and lipase levels (c, error bars refer to 10–90% of the data), and histological score (e, error bars refer to mean with 95% CI). Similarly, ICR mice receiving long-term administration were assessed by group pathology (f), amylase and lipase (g, error bars refer to 10–90% of the data), and histological score (d, error bars refer to mean with 95% CI). Pathological images were selected from representative HE-stained images. Amylase and lipase results are presented as box plots (right), combined with data distributions (left) and displayed on a dual y-axis.
